# Attenuated Expression of Apoptosis Stimulating Protein of p53-2 (ASPP2) in Human Acute Leukemia Is Associated with Therapy Failure

**DOI:** 10.1371/journal.pone.0080193

**Published:** 2013-11-27

**Authors:** Marcus M. Schittenhelm, Barbara Illing, Figen Ahmut, Katharina Henriette Rasp, Gunnar Blumenstock, Konstanze Döhner, Charles D. Lopez, Kerstin M. Kampa-Schittenhelm

**Affiliations:** 1 University Hospital Tübingen, Department of Hematology, Oncology, Rheumatology, Immunology and Pulmology, Tübingen, Germany; 2 Department of Clinical Epidemiology and Applied Biometry, Eberhard Karls University Tübingen, Germany; 3 University Hospital Ulm, Department of Internal Medicine III, Ulm, Germany; 4 Department of Medicine, Division of Hematology and Medical Oncology, Oregon Health & Science University and The OHSU Knight Cancer Institute, Portland, Oregon, United States of America; University of Saarland Medical School, Germany

## Abstract

Inactivation of the p53 pathway is a universal event in human cancers and promotes tumorigenesis and resistance to chemotherapy. Inactivating p53 mutations are uncommon in non-complex karyotype leukemias, thus the p53-pathway must be inactivated by other mechanisms. The Apoptosis Stimulating Protein of p53-2 (ASPP2) is a damage-inducible p53-binding protein that enhances apoptosis at least in part through a p53-mediated pathway. We have previously shown, that ASPP2 is an independent haploinsufficient tumor suppressor *in vivo*. Now, we reveal that ASPP2 expression is significantly attenuated in acute myeloid and lymphoid leukemia – especially in patients with an unfavorable prognostic risk profile and patients who fail induction chemotherapy. In line, knock down of ASPP2 in expressing leukemia cell lines and native leukemic blasts attenuates damage-induced apoptosis. Furthermore, cultured blasts derived from high-risk leukemias fail to induce ASPP2 expression upon anthracycline treatment. The mechanisms of ASPP2 dysregulation are unknown. We provide evidence that attenuation of ASPP2 is caused by hypermethylation of the promoter and 5′UTR regions in native leukemia blasts. Together, our results suggest that ASPP2 contributes to the biology of leukemia and expression should be further explored as a potential prognostic and/or predictive biomarker to monitor therapy responses in acute leukemia.

## Introduction

The p53 pathway is well known as a central player in the cellular stress response and tumor suppression. Depending on the nature of the stress, the outcome of p53 activation can range from cell cycle arrest and DNA repair to senescence, autophagy and apoptosis. Not surprisingly, inactivation of the p53 pathway is a universal event in human cancers and p53 is one of the most highly mutated genes with greater than 60% of human malignancies harboring inactivating p53 mutations (reviewed by Vousden and Prives[1]; UMD TP53 mutation database at http://p53.fr/ [2]).

In some hematopoietic malignancies, inactivating mutations of p53 are involved with chromosomal instability and progression towards acute leukemia, such as complex karyotype myelodysplastic syndromes[3], [4], [5], [6] and chronic phase Philadelphia-chromosome positive chronic myeloid leukemia (CML)[7]. Moreover, chromosomal aberrations of the long arm of chromosome 17 (locus of p53) or inactivating p53 mutations impede cancer therapies, such as fludarabine-based chemotherapy in CLL[8], BCR/ABL-targeted therapies in CML[9] – as well as induction chemotherapy in AML[10].

However, in most cases of de novo acute leukemia, p53 mutations or chromosomal aberrations of chromosome 17 are uncommon, but primarily associate with therapy-related acute myeloid leukemias[11] and MDS-related complex karyotype leukemia[10], [12], [13]. We therefore speculated that in acute leukemia the p53 pathway may be altered by other means besides mutation. However the molecular mechanisms that inactivate the p53 pathway in acute leukemia remain unclear.

Apoptosis Stimulating Protein of p53-2 (ASPP2), also referred to as 53BP2L, encoded by *TP53BP2* enhances damage-induced apoptosis at least in part through a p53-mediated pathway[14]. Mouse models targeting ASPP2 using homologous recombination demonstrate that ASPP2 is a haploinsufficient tumor suppressor[15], [16]. Indeed, ASPP2 expression is frequently suppressed in a variety of human cancers, such as breast cancer[14] and lymphoma subtypes, where low ASPP2 mRNA expression levels are associated with biologically more aggressive lymphoma subtypes and with poor clinical outcome[17]. ASPP2 is also a damage-inducible protein. Depending on cell context and type of stress, ASPP2 levels typically increase via transcriptional and/or posttranslational mechanisms after cellular damage[18], [19]. Thus, the complex regulation of ASPP2 expression may provide important prognostic or predictive clinical information. However, to date there have been no studies examining ASPP2 expression in acute leukemia.

In this report, we now demonstrate that lower ASPP2 mRNA and protein expression levels are statistically significantly associated with clinical unfavorable disease and early chemotherapy-induction failure in de novo as well as secondary acute myeloid and lymphoid leukemia. Moreover, ASPP2 siRNA knockdown in leukemia cell lines and *ex vivo* cultured patient derived primary leukemic blasts results in resistance to anthracycline-induced apoptosis.

Our findings provide evidence that ASPP2 plays a role in the biology of acute leukemia and might serve as a biomarker to risk stratify patients and monitor therapy responses.

## Design and Methods

### Cell lines

The promyelocytic AML cell line HL60 was purchased from the Leibniz Institute-German Collection of Microorganisms and Cell Cultures (DSMZ), Germany. The acute T-cell lymphoblastic leukemia cell line Jurkat was a gift from Dr. Salih, University of Tübingen. The CML blast crisis cell line K562 was a generous gift of Dr. Lopez, Oregon Health and Science University, Portland, OR. The core binding factor leukemia cell line Kasumi-1 was obtained from the German Collection of Microorganisms and Cell Cultures (DSMZ).

Cells were cultured in RPMI 1640, supplemented with 10% fetal bovine serum (GIBCO/Invitrogen, Darmstadt, Germany), 1% penicillin G (10,000 units/mL) and streptomycin (10,000 µg/mg) and 2 mmol/L l-glutamine (GIBCO/Invitrogen, Darmstadt, Germany or Biochrom AG, Berlin, Germany).

### Antibodies and reagents

An anti-ASPP2 isoform 1/2 monoclonal mouse antibody (Sigma, MO) targeting an epitope within aminoacids 691–1128 was used at a 1∶1,000 to 1∶250 dilution. Anti-tubulin antibody was used as a loading control (Cell Signaling, Danvers, MA).

For flow cytometry studies fluorescent dye-conjugated (AlexaFluor®) secondary goat anti-mouse was used according to standard protocols (Cell Signaling, Danvers, MA).

Daunorubicin was obtained from the University of Tübingen Hospital Pharmacy and dissolved in DMSO to create a 1,77 mmol/L stock solution and stored at −20°C.

### Isolation of bone marrow and peripheral blood mononuclear cells

Bone marrow aspirate and peripheral blood samples from patients with diagnosed acute leukemia (patient characteristics are provided in Tables 1 and 2) or healthy volunteers (bone marrow or blood donors) were collected in 5000 U heparin after written informed consent and approval of the ethics committee of the Universities of Tübingen and Ulm, respectively. Mononuclear cells were isolated by Ficoll Hypaque density gradient fractionation[20], [21]

**Table 1:** Patient Characteristics (mRNA Assay): Good-Risk Cohort.

Pt.Nr.	Specimen	Age	Gender	Leukemia Subtype	Prognostic	Induction-	Response	ASPP2/GAPDH
				*acc. to risk factors*	Risk Group	therapy	*after 1st Induction*	*mRNA Expression*
299	peripheral blood	55	male	CBFL	good	yes	CR	0.81
295	peripheral blood	61	male	CBFL (*KIT* D816V+)	good/intermediate	yes	CR	0.88
281	peripheral blood	43	male	CBFL	good	yes	PR	0.96
233	peripheral blood	28	female	CBFL	good	yes	CRi	1.20
231	peripheral blood	41	female	CBFL	good	yes	CR	1.51
293	peripheral blood	55	female	CBFL	good	yes	PR	1.60
167	bone marrow	39	male	APL, therapy-related	good/intermediate	yes	CR	2.16
46	bone marrow	75	female	CBFL	good	yes	CR	2.58
322	bone marrow	57	male	AML (mutant-*NPM1*+)	good	yes	CR	2.75
521	peripheral blood	45	female	CBFL	good	yes	PR	2.89
349	peripheral blood	39	male	CBFL	good	yes	CR	3.32
92	peripheral blood	69	female	AML (mutant-*NPM1*+)	good	yes	CR	3.78
317	peripheral blood	19	male	CBFL (*KIT* D816Y+)	good/intermediate	yes	CR	4.57
157	peripheral blood	55	female	CBFL, therapy-related, paravertebral chloroma	good/intermediate	yes	PR	5.25
87	peripheral blood	48	male	CBFL	good	yes	CR	5.27
257	peripheral blood	66	male	CBFL	good	yes	CRi	5.59
221	peripheral blood	46	female	CBFL	good	yes	CR	5.62
279	peripheral blood	46	female	CBFL	good	yes	CRi	6.15
378	bone marrow	57	female	CBFL	good	yes	CR	7.17
275	peripheral blood	19	male	CBFL	good	yes	CR	7.68
361	peripheral blood	52	male	CBFL (*KIT* D816V+)	good/intermediate	yes	CR	8.29
85	peripheral blood	35	male	CBFL	good	yes	CR	9.96
38	peripheral blood	70	male	APL	good	yes	CR	15.99
305	peripheral blood	33	female	CBFL	good	yes	CR	97.11

The prognostic good-risk population is segregated from the total cohort according to ELN-guidelines^22^; this includes Core-binding Factor Leukemia (CBFL), Acute Promyelocytic Leukemia (APL) and Nucleophosmin1-mutated AML.

The CBFL group includes KIT-mutated cases, which have an adverse prognosis in some studies^38^. Induction therapy was based on anthracycline plus cytarabine chemotherapy. Complete Remissions include cases with complete remission with incomplete hematopoietic recovery (CRi).

**Table:** Table 2. Patient Characteristics (mRNA Assay): Higher-Risk Cohort.

Pt.Nr.	Specimen	Age	Gender	Leukemia Subtype	Induction-	Response	ASPP2/GAPDH
				according to risk factors	therapy	after 1st Induction	mRNA Expression
234	bone marrow	65	female	sAML (MDS)	yes	refractory	0.03
135	bone marrow	64	male	biphenotypic AML/AUL	yes	refractory	0.09
66	peripheral blood	41	female	AML (mutant-*FLT3*+)	yes	refractory	0.44
67	peripheral blood	73	female	sAML (MDS)	no (palliation)	n/a	0.45
64	peripheral blood	49	male	AML (WBC>100 000/µl)	yes	early death during induction	0.52
368	bone marrow	77	female	tAML/complex karyotype AML	yes	CR	0.61
109	bone marrow	75	male	sAML (MDS)	yes	PR	0.71
11	peripheral blood	28	male	biphenotypic/AUL	yes	CR	1.09
24	peripheral blood	49	male	AML (mutant-*MLL*+)	yes	CR	1.33
60	bone marrow	57	female	complex karyotype AML	yes	PR	1.43
25	bone marrow	44	male	AML (mutant-*FLT3*+)	yes	CR	2.41
74	peripheral blood	67	male	complex karyotype AML	yes	refractory	2.84
80	peripheral blood	45	male	AML (mutant-*FLT3*+)	yes	CR	3.01
27	bone marrow	45	male	AML (mutant-*MLL*+)	yes	CR	3.07
273	peripheral blood	66	male	AML (mutant-*FLT3*+)	yes	PR	3.10
48	peripheral blood	62	female	Ph+ALL/biphenotypic AML	yes	CR	3.26
284	bone marrow	39	female	AML (mutant-*FLT3*+)	yes	PR	3.63
236	bone marrow	41	female	tAML	yes	PR	3.84
23	bone marrow	73	male	sAML (MDS)	no (palliation)	n/a	4.34
8	peripheral blood	67	female	AML (mutant-*FLT3*+)	yes	PR	7.95
39	peripheral blood	50	female	sAML (MDS)	yes	CR	10.16
22	bone marrow	62	male	Ph+ALL/biphenotypic AML	yes	CR	10.23
36	bone marrow	72	male	biphenotypic AML/AUL	yes	refractory	17.96
3	bone marrow	68	male	sAML (MDS)	no (palliation)	n/a	19.87
110	bone marrow	75	male	sAML (MDS)	no (palliation)	n/a	24.79
99	peripheral blood	85	male	AML (WBC>100 000/µl)	no (palliation)	n/a	31.46

The prognostic higher-risk cohort includes AML with leukocytosis >100,000/microliter, secondary and complex karyotype AML (from MDS), therapy-related AML, biphenotypic and undifferentiated leukemia, Philadelphia-chromosome-, FLT3- or MLL-mutated myeloid or lymphoid leukemia.

### Polymerase Chain Reaction (PCR)

mRNA was isolated and reverse transcribed using standard techniques and a RNeasy® RNA purification kit (Qiagen, Hilden, Germany). ASPP2 mRNA expression levels, relative to GAPD as the housekeeping gene, were determined by qRT-PCR Roche® LightCycler Technology (Roche, Basel, Switzerland). The ASPP2 primer set was as follows: sense 5′-aacccggcgagagtgatt-3′, antisense 5′-tggccaacatgatgaaactc-3′.

### Statistical analysis

ASPP2 mRNA expression data was normalized to a donor control. The normalized ASPP2 mRNA expression levels were highly positively skewed and are reported with the median and range on a logarithmic scale. Samples were compared primarily using the non-parametric Wilcoxon rank-sum test and Kruskal-Wallis test, respectively. Similar results were obtained with one-way ANOVA and the two-sample t test after log-log-transformation to meet the test assumptions (not shown). This part of the analysis was done with the JMP® 10.0 statistical software (SAS Institute, Cary, NC, USA).

### Flow cytometry based ASPP2 protein expression

Cells were fixed and permeabilized using the Fix & Perm® Fixation and Permeabilization kit (ADG-An der Grub Bioresearch, Kaumberg, Austria). The unlabeled primary ASPP2 antibody was added in a 1∶1000 dilution to the cell suspension and incubated for 1 hour at room temperature followed by PBS washing and resuspension. Fluorescent dye-conjugated (AlexaFluor®) secondary goat anti-mouse was added in a 1∶10 000 dilution and cells were incubated for 30 min at room temperature. After rinsing and resuspension, ASPP2-protein expression levels were assayed using a FACScalibur® flow cytometer loaded with CellQuest® analysis software (BD, Heidelberg, Germany).

### Western immunoblotting

Protein from cell lysates (75 to 200 µg protein) was used for whole cell protein analysis after denaturing by Western immunoblot assays using a BioRad Criterion system (protein separation by SDS-PAGE in 10% polyacrylamide gels followed by electroblotting onto nitrocellulose membranes). Nonspecific binding was blocked by incubating the blots in nonfat dry milk. Primary ASPP2 antibody was incubated for one hour or over night, followed by several washes of Tris-buffered saline (TBS) containing 0.005% Tween 20. iRDye secondary antibody was applied for 30‘, followed by several washes and antibody-reactive proteins were detected using a LI-COR Odyssey® fluorescence optical system (LI-COR Biosciences, Lincoln, NE).

### siRNA ASPP2 knockdown in human acute myeloid cell lines

Six small interfering (si)RNA constructs (Stealth RNAi™ siRNA, Invitrogen, CA) targeting ASPP2 at #1: 5′ gcuguggaagaagaaggcagcucua 3′ [Exon 9 sense and antisense]; #2: 5′ gcgggaugcucagguugcaaauaaa 3′ [Exon 12/13 sense and antisense] and #3: 5′ cagaggg tcctaatggg ccaaatat 3′ [Exon 14 sense and antisense] were used in an assay to silence ASPP2 expression. The constructs or a scrambled siRNA control were lipofected into cells using a lipofectamine 2000®-based protocol (Invitrogen, CA). Briefly, 5×10^5^ cells/well were plated in a 24-well plate and cultured in medium containing serum without antibiotics. Constructs were diluted in serum-free medium and mixed with a lipofectamine. The mix was then added to the cell suspension followed by incubation at 37°C. Transfection efficacy was independently validated by siGLO transfection indicator (Dharmacon).

### Apoptosis assays

Apoptosis was analyzed using standard Annexin V-based assays (Immunotech, Marseilles, France) and a FACScalibur flow cytometer using CellQuest analysis software (BD, Heidelberg, Germany)[21].

## Results

### ASPP2 mRNA expression is significantly attenuated in acute leukemia patients

**Figure 1:**
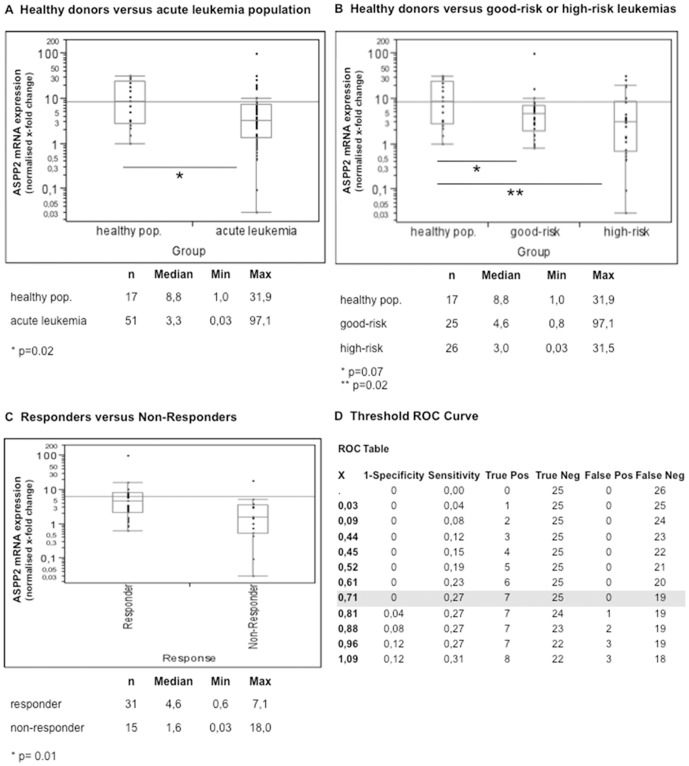
ASPP2 mRNA expression in acute leukemia. qRT-PCR based mRNA expression levels are displayed after normalizing to a healthy blood donor (set as 1) on a logarithmic scale. Cohort analysis reveals significant lower ASPP2 levels for an acute leukemia population compared to a healthy peripheral blood and bone marrow donor cohort (A). Comparison of prognostic risk groups confirms lower ASPP2 expression levels for the good-risk as well as higher-risk cohort when compared to a healthy donor population – whereas attenuated ASPP2 expression levels are more pronounced and statistically significantly different for the higher-risk cohort (B). Analysis of therapy responders (i.e. achievement of complete remission after one cycle of induction chemotherapy) demonstrates significantly lower ASPP2 levels for the therapy-failure population when compared to the responder cohort (including good-/higher-risk pts.) (C). ROC curve analysis defining the ideal threshold to distinguish a definite non-responding sub-population is shown in figure 1D (i.e. patients with attenuated ASPP2 expression levels ≤0.8 are likely not to respond to induction chemotherapy (with no single falsely positive tested patient at this threshold). P-values are provided as indicated by an asterix. Patient characteristics, including definitions of the prognostic risk groups, are summarized in Table 1 and 2.

Inactivation of the p53 pathway is a universal event in human cancer. However, the majority of de novo acute leukemias are wildtype for p53. Since (i) ASPP2 binds p53 and stimulates p53-dependent apoptosis and (ii) attenuated ASPP2 expression correlates with poor clinical outcome in aggressive DLBC lymphomas[17], we hypothesized that attenuated ASPP2 expression is related to poor clinical outcomes in AML. Compared to 17 healthy blood (n = 8) or bone marrow (n = 9) donors, we found by qRT-PCR significantly lower median ASPP2 mRNA expression levels in leukemic blasts collected sequentially from 51 consented patients with newly diagnosed acute myeloid or lymphoid leukemia (median normalized expression levels 3.3 vs. 8.8). This difference was statistically significant using a Wilcoxon rank sum-test (p = 0.02) (Figure 1A; patient characteristics are provided with Table 1 (good-risk cohort[22]) and Table 2 (higher-risk cohort)). Strikingly however, in contrast to the healthy donor samples, where ASPP2 expression levels clustered within a range of 1 and 31, ASPP2 mRNA expression in acute leukemia samples revealed wide expression variance ranging from 0.03 to 97–fold. Subcohort analysis of bone marrow aspirates (n = 26) versus peripheral blood samples (n = 30), derived from consented patients with newly diagnosed acute leukemia, did not reveal significant differences in ASPP2 expression levels (p = 0.75; median_bone marrow_ 3.3, *min. 0.0, max. 24.8*; median_peripheral blood_ 3.5, *min. 0.4, max. 31.5*; data not shown).

When the prognostic good-risk leukemia patients (adapted to the European LeukemiaNET Genetic Risk Classification[22]) were segregated from all other patients (summarized as “higher-risk cohort”, Tables 1 and 2), the good-risk cohort trended to lower ASPP2 expression levels when compared to healthy donors (medians 4.6 versus 8.8). However, this difference failed significance barely (p = 0.07) due to marked differences in expression variance within the good-risk population. In contrast, within the higher-risk cohort we found very low ASPP2 expression in a distinct proportion of patients compared to both healthy donors and to the good-risk population. Despite high overall variance of ASPP2 expression in higher-risk patients (range 0.03–31), median expression levels were statistically significantly lower compared to the healthy donor cohort (p = 0.02) (Figure 1B).

To determine if ASPP2 levels were different in responders who achieved complete remission after one cycle of induction chemotherapy (independent of clinical prognostic stratification) compared to non-responders, we used a two sample rank sum-testing to demonstrate significantly lower ASPP2 expression levels in the non-responder cohort (p = 0.01) (Figure 1C). However, highly variable expression levels within the non-responder group limited our ability to segregate the responder cohort from all non-responders. Interestingly, 27 percent of the non-responding cohort displayed ASPP2 levels less than 0.8. Such low levels were not observed in any patients in the good-prognostic cohort (0%) nor in higher-risk patients achieving complete remission after one cycle of induction chemotherapy (0%). Moreover, receiver operating characteristic (ROC) analysis determined an optimal threshold level of ≤0.8 for safely detecting patients who are likely to fail induction chemotherapy – with no patient of the responder cohort classified as false-positive at this cut-off value (figure 1D).

It was previously suggested that ASPP2 protein levels are controlled – at least to some extend – post-translationally (e.g. via ubiquitination)[19]. To evaluate, whether mRNA expression levels correlate with protein expression levels, we performed a western immunoblot assay using several native leukemia samples of the good-risk cohort. Indeed, at least in their extremes, mRNA and protein expression levels correlated in native leukemia specimens – with samples expressing very low ASPP2 mRNA levels displaying low to absent protein expression levels (see Figure S1 with the online version of the manuscript).

Together, these results demonstrate, that ASPP2 expression levels are attenuated in a subset of acute leukemias. Attenuation of ASPP2 is especially pronounced in patients classified into the prognostic higher-risk cohort. Within this cohort, ASPP2 expression levels ≤0.8 predict patients who fail to achieve early complete remission after first induction chemotherapy.

### Attenuation of ASPP2 expression inhibits chemotherapy-induced apoptosis in leukemia cells *in vitro*

**Figure 2:**
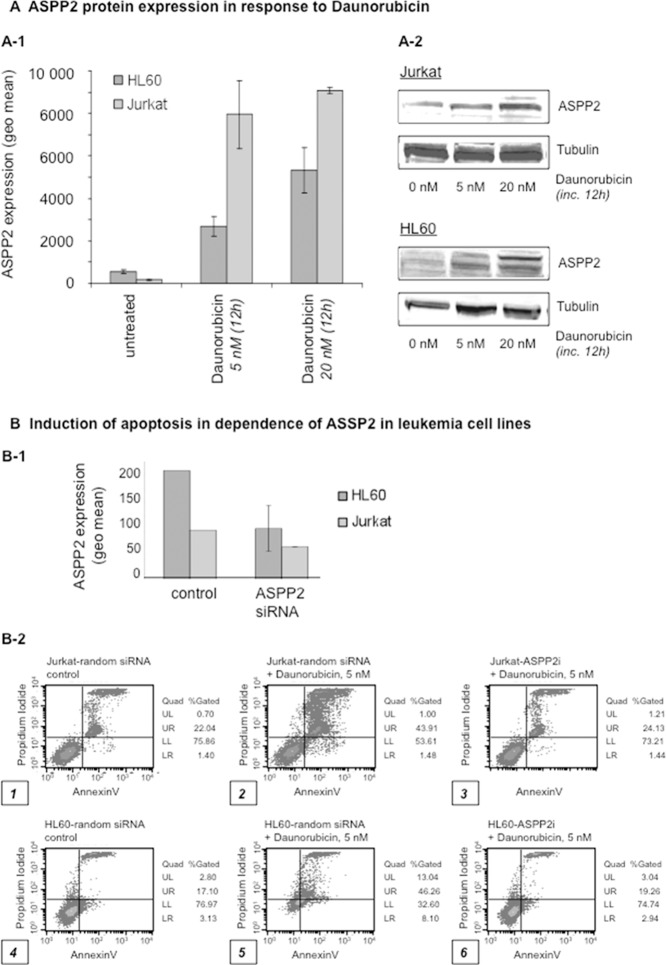
ASPP2 is induced upon anthracycline exposure to promote apoptosis. (A) Intracellular ASPP2 protein expression levels increase upon daunorubicin treatment in a dose-dependent manner in the acute myeloid leukemia HL60 cell line and the T-lymphoblastic leukemia Jurkat cell line. A flow cytometry based assay is shown (A-1) which is confirmed by a Western immunoblot (A-2). (B) Lipofection of Jurkat and HL60 leukemia cell lines with specific ASPP2 siRNA or random siRNA as negative controls was performed. Cells were treated with daunorubicin (5 nM) for 48 hours and induction of apoptosis was measured using an Annexin V-based assay and flow cytometry.

Since ASPP2 expression can be induced by cellular damage to promote apoptosis[19], [23] and low ASPP2 levels are associated with poor-risk AML with early CR-failure (Figure 1), we wished to explore if ASPP2 levels correlate with leukemia cell sensitivity to daunorubicin-induced apoptosis. To test this, we utilized the promyelocytic leukemia cell line HL60 and the lymphoblastic leukemia Jurkat cell line. We found induction of that ASPP2 protein levels to be induced 12 hours after exposure to daunorubicin in these cell lines using a flow cytometry-based intracellular immunostaining method (Figure 2A). Analysis of intracellular ASPP2 protein expression using flow cytometry-based immunophenotyping has not been previously described and we validated the assay by Western immunoblotting (sub-figure 2A-2) as described in previous reports(24). To determine if ASPP2 modulated daunorubicin-induced apoptosis (as determined in an annexin V-based assay), we utilized siRNA to silence ASPP2 expression in these leukemia cell lines (Figure 2B). Confirmation of ASPP2 siRNA knockdown is provided with sub-figure 2B-1. We found that attenuation of ASPP2 expression by two different siRNAs inhibited daunorubicin-induced apoptosis in Jurkat (panel 1-3) and in HL60 (panels 4-6) cell lines, compared to control siRNA (panels 1 and 4) (sub-figure 2B-2). These results demonstrate that ASPP2 modulates chemotherapy-induced apoptosis – which is consistent with our clinical observations that low ASPP2 levels are related to chemotherapy resistance and poor prognosis in AML patients.

### ASPP2 protein expression in fresh harvested primary leukemia blasts is associated with clinical outcome

**Figure 3:**
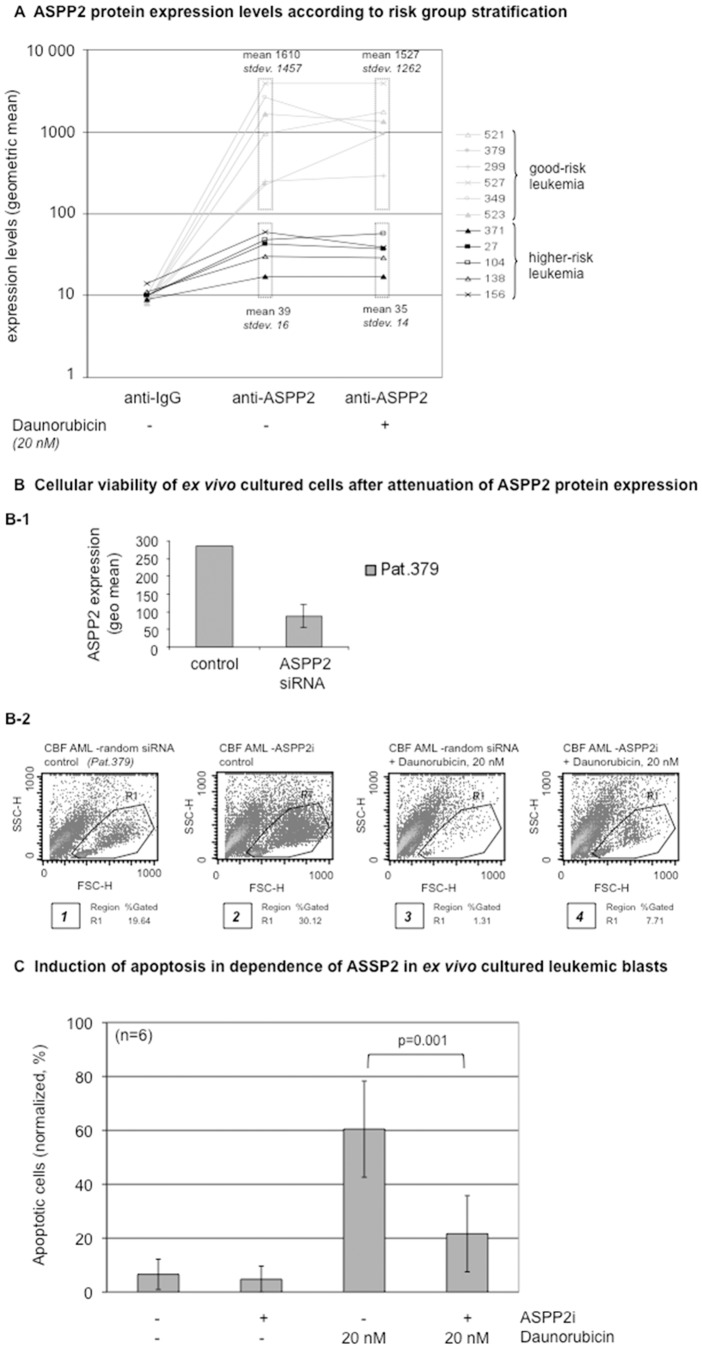
ASPP2 expression in fresh harvested primary acute leukemic blasts treated ex vivo. (A) Intracellular ASPP2 protein expression measured by flow cytometry in primary leukemia blasts derived from 11 patients is shown. IgG control represents background levels. Additionally, induction of ASPP2 expression upon daunorubicin (20 nM) exposure for 24 hours is determined on *ex vivo* cultured cells (right column). Good-risk versus higher-risk prognostic cohorts are indicated as defined in Table 3. (B) Cellular viability in ASPP2-siRNA knocked down primary leukemic blasts after daunorubicin treatment for 72 hours is determined by FSC/SSC flow cytometry. R1 gate set to indicate viable cells. ASPP2 siRNA knockdown (B-1) was validated against a random siRNA control. (C) A bar diagram summarizing apoptosis assays derived from 4 cell lines (HL60, Kasumi-1, Jurkat, K562) and 2 native core binding factor leukemia samples (pts. 378 and 521) is shown. Cells were pretreated with ASPP2 siRNA as indicated. Application of 20 nM daunorubicin was set up for 48 hours and induction of apotosis was measured in an annexin V-based assay. ASPP2-interference leads to highly significant impairment of proapoptotic effects as demonstrated in a paired student's t-test (p = 0.001).

Since ASPP2 mRNA expression associated with risk subgroups in patients with acute leukemia (Figure 1), we wished to examine ASPP2 protein expression in patient-derived primary leukemic blasts using our flow cytometry-based method (Figure 3). After Ficoll hypaque-extraction, leukemic blasts were fixed and permeabilized to detect intracellular ASPP2 protein levels. Clinical characteristics are summarized in Table 3. We analyzed 11 evaluable patients and found high ASPP2 expression in 6 patients (with a geometric mean at an average of 1610 SD 1457); and lower ASPP2 protein expression in five patients (geometric mean 39.4, standard deviation 16.3). As expected, the higher expressing and lower ASPP2 expressing cohorts segregated into good versus higher-risk prognostic subgroups – with the higher-risk subgroup associating with lower ASPP2 protein expression levels and the good-risk subgroup linking to higher ASPP2 expression (Figure 3A - middle column).

**Table:** Table 3. Patient Characteristics (Protein Assay): Higher vs. Good-Risk Cohorts.

Pt.Nr.	Specimen	Age	Gender	Leukemia Subtype	Prognostic Risk Group	Response towards Chemotherapy (Cx)	Protein Expression (geo mean, basal levels)	Protein Expression (geo mean, post daunorubicin)
				*acc. to risk factors*			*IgG control set <10*	*IgG control set <10*
27	bone marrow	48	male	AML (mutant-*MLL*+)	high	CR after 1^st^ induction Cx	43	37
156	bone marrow	78	male	sAML (MDS)	high	PR after 1^st^ induction Cx	59	39
138	peripheral blood	85	male	AML (WBC>100 000/µl)	high	palliation therapy	30	29
104	peripheral blood	65	male	Ph+ALL	high	palliation therapy	48	57
371	peripheral blood	32	female	Ph+ALL	high	CR after 1^st^ induction Cx	17	17
299	peripheral blood	55	male	CBFL	good	CR after 1^st^ induction Cx	249	288
349	peripheral blood	39	male	CBFL	good	CR after 1^st^ induction Cx	2630	932
379	peripheral blood	56	female	CBFL	good	CR after 1^st^ induction Cx	227	942
521	peripheral blood	45	female	CBFL	good	PR after 1^st^ induction Cx	959	1753
523	peripheral blood	51	female	CBFL	good	CR after 1^st^ induction Cx	1665	1343
527	peripheral blood	65	male	APL	good	CR after 1^st^ induction Cx	3930	3905

For prognostic risk group definitions, see Table 1.

To determine if ASPP2 protein could be induced in primary AML blasts, we treated *ex vivo* cultured blasts with 20 nM of daunorubicin and measured ASPP2 protein expression after 12 hours. We found that patients in the higher-risk cohort demonstrated lower baseline ASPP2 protein levels that did not significantly increase after daunorucibicin (Figure 3A - right column). In contrast, the good-risk patients demonstrated higher baseline ASPP2 protein levels, with some of the samples showing an increase in ASPP2 protein expression after daunorubicin (Figure 3A - right column). These findings suggest that baseline ASPP2 protein levels and resistance to damage-induction may be associated with clinical prognosis and induction failure.

### Attenuation of ASPP2 expression inhibits chemotherapy-induced apoptosis in *ex vivo* cultured primary native blasts

To demonstrate whether ASPP2 can modulate sensitivity to daunorubicin-induced cell death in patient-derived leukemic blasts, we attenuated ASPP2 expression using siRNA in freshly isolated blasts derived from a good-risk subgroup patient (#379) that expressed high ASPP2 protein levels (Figure 3A). After knockdown of ASPP2 expression with siRNA (Figure 3B-1), we found that these primary blasts were more resistant to daunorubicin-induced cell death when compared to control siRNA treated blasts as measured in a flow cytometry based cell viability assay (Figure 3B-2, panel 3 and 4). Intriguingly, siRNA transfection increased viability of *ex vivo* cultured blasts in the absence of damage as indicated by an increase in the percentage of viable cells compared to a random siRNA-transfected, untreated cell sample (Figure 3B, panel 1 versus panel 2). This is consistent with attenuated ASPP2 expression promoting cell survival in primary human leukemic blasts in *ex vivo* culture conditions by impairment of ASPP2-mediated control of programmed cell death.

We further set up an annexin V-based apoptosis assay to statistically evaluate the proapoptotic efficacy in dependence of ASPP2 in leukemia cell lines as well as native leukemia samples. Irrespective of the origin of cells (*in vitro* cell line models or *ex vivo* native leukemia blasts), ASPP2-interference lead to abrogation of proapoptotic effects induced by anthracycline therapy (figure 3C). This observation was highly significant in a paired student's t-test (p = 0.001), when compared to assays with the parental samples (without ASPP2 siRNA pretreatment).

Together, these results suggest that ASPP2 expression is important for modulating the response of acute leukemia blasts to chemotherapy.

## Discussion

Acute leukemias remain difficult to treat and many patients still die of their disease. Achieving an early therapy response is crucial – but it remains difficult to identify patients who will fail first-line induction chemotherapy. Currently, success of induction therapy is monitored by examination of day +15 and day +21-28 bone marrow aspirates in order to assess for restoration of normal hematopoiesis and peripheral blood counts to inform therapeutic decisions. Thus, there is a critical need to improve our ability to identify responsive versus non-responsive disease early during the course of induction therapy. Biomarkers, which predict and/or monitor therapeutic success prior to or during therapy may greatly improve therapeutic strategies − especially for identifying early induction failures. This would potentially permit dose and/or frequency intensification of chemotherapy regimens, addition of chemotherapeutics, antibodies or small molecules as well as referral to rapid allogeneic transplantation strategies.

In this report, we identify ASPP2 as a potential biomarker for early chemotherapy induction failure and poor prognosis. ASPP2 is a damage-inducible p53 binding protein that stimulates p53-dependent as well as p63- and p73-dependent apoptosis[14], [24], [25]. Attenuation of ASPP2 expression promotes both spontaneous and damage-induced tumors in mouse models[15], [16], and is associated with cancer development and poor clinical outcome in human lymphoma[17]. Interestingly, mounting evidence is also accumulating showing that ASPP2 promotes p53-independent cell death and growth inhibition[26], [27], [28], [29], [30], [31], [32]. Our findings suggest that attenuated ASPP2 expression is a mechanism to promote resistance to chemotherapy in acute human leukemias. How attenuation of ASPP2 expression modulates p53-dependent and/or p53-independent pathways in acute leukemia remains to be elucidated.

In this study we analyzed ASPP2 mRNA expression in freshly isolated blasts from 51 patients with acute myeloid or lymphoid leukemia and found a wide range in expression levels (Figure 1). Notably however, ASPP2 expression in these acute leukemic blasts was significantly lower than ASPP2 expression from a healthy donor population. Intriguingly, when we further analyzed the wide range in ASPP2 expression in blasts with respect to clinical prognostic risk groups, we found that very low relative ASPP2 levels segregated into a higher-risk clinical group that had failed anthracycline plus cytarabine-based initial induction chemotherapy. Importantly, these findings were statistically significant using the non-parametric Wilcoxon rank-sum test and Kruskal-Wallis test, respectively.

Because ASPP2 is a damage-inducible protein, we wanted to determine whether chemotherapy-induced ASPP2 induction in freshly isolated acute leukemic blasts could further identify patients with high-risk clinical characteristics using a rapid method that could be adapted into clinical use easily. To do this, we first developed a flow cytometric-based method to quantify induction of ASPP2 protein expression using established leukemic cell lines (Figures 2). Importantly, we also used this method to demonstrate that ASPP2 knockdown in these leukemic cell lines promoted resistance to chemotherapy-induced cell death. Using our flow-based method on freshly isolated blasts treated *ex vivo* with daunorubicin, we found that ASPP2 protein expression could be induced in some good-risk patients' blasts compared to no induction in any higher-risk patients (Figures 3). Although the sample size tested for protein expression was not large enough to draw statistical conclusions, this proof of principle experiment is consistent with a role for the lack of ASPP2 damage-induction[23] playing a role in resistance to chemotherapy in human leukemia. Importantly, we anticipate using our methodology to rapidly analyze primary fresh isolated leukemic blasts from patients in a prospective manner. In this context, we quantified ASPP2 mRNA expression in patients undergoing induction chemotherapy and found an increase in ASPP2 levels on day 3 post-induction therapy (preliminary data not shown). Thus, we have recently launched a prospective analysis of ASPP2 expression of leukemia blasts isolated from patients prior to, during and after induction chemotherapy.

Our findings that knockdown of ASPP2 expression in both established and primary leukemic cell lines inhibits chemotherapy-induced apoptosis (Figures 2 and 3) demonstrates the functional importance of ASPP2 in acute leukemia response to therapy. To what extent this involves p53-dependent, as well as p53-independent mechanisms, remains unknown. However, given the complexities of the cellular response to genotoxic-damage, it is likely that multiple mechanisms will play a role in these processes. Notably, whereas the human leukemia sample used for siRNA knock-down (Figure 3B) was confirmed to be p53-wildtype (data not shown) - HL60 and Jurkat leukemia lines (Figure 2), are known to harbor p53 mutations [33], [34]. This tantalizingly suggests that ASPP2 can also modulate apoptosis via p53-independent pathways in leukemia cells. Whether p53 family members[25] (or other factors) may also play a role remains to be determined and is under investigation.

Whenever collecting fresh tumor samples, it remains possible that variability in handling, storage and preparation of cells may interfere with our interpretation of ASPP2 expression, since ASPP2 expression is stress-inducible[18]. However, low ASPP2 expression levels in the presence of cellular stress argue for a disabled ASPP2 stress response. This is consistent with our statistically significant clinical correlations, which together with our functional siRNA experiments, argues for a central role of ASPP2 in the therapeutic response to induction chemotherapy in acute leukemia.

ASPP2 damage-induction occurs at both transcriptional and post-transcriptional levels[19], [23], [35]. However, the mechanism by which ASPP2 damage-induction occurs in primary human leukemic cells remains to be determined. Sequence analysis of the published ASPP2 promoter region[35], and the entire open reading frame from ASPP2 low- and ASPP2-high-expressing isolated primary AML blasts did not reveal mutations or genomic changes (data not shown). Interestingly however, a gDNA methylation screen including five patients with higher-risk myeloid or lymphoid leukemia revealed methylation at 15/20 probes spanning the ASPP2 gene from the transcription start site (TSS) to the 3′ untranslated region (UTR) (see Figure S2). Methylation patterns were particular high in the 5′UTR region and immediate downstream regions, which in ASPP2 is encoded by most of exon 1. This is in particular noteworthy as a recent report found that DNA methylation downstream of the TSS, in the region of the first exon, is most tightly linked to transcriptional silencing compared to all other regions[36]. These findings are further supported by a report demonstrating epigenetic suppression of ASPP2 expression by gDNA methylation as suggested in hepatitis B-associated hepatocellular carcinoma[37]. Thus, it is tempting to speculate that similar epigenetic mechanisms may silence ASPP2 expression in leukemia and that the recent success of demethylating agents in these diseases may be mediated in part by restoration of ASPP2 expression[36]. However, this remains to be formally tested and needs further investigation.

In summary, we now provide proof-of-principle data suggesting that ASPP2 may affect the therapeutic response to chemotherapy in acute leukemia. This suggests that monitoring of ASPP2 expression in patient blasts during induction chemotherapy might eventually be clinically valuable. For example, ASPP2 expression could be a predictive biomarker for the early assessment of therapeutic responses in acute leukemia prior to and during induction chemotherapy. This could potentially help to identify patients who will most likely fail induction chemotherapy - thus allowing early therapy intensification/modification to improve outcome and survival. Thus, prospective clinical studies to further define the role of ASPP2 as a biomarker in acute leukemia – especially in the context of other prognostic markers – are warranted.

## Supporting Information

Figure S1 ASPP2 mRNA levels translate into protein expression levels. Protein lysates of native leukemia samples with a good-prognostic profile (patient characteristics are provided with Table 1) are immunoblotted to detect ASPP2 protein levels. The MCF-7 breast cancer cell line is used as a positive control to detect ASPP2 protein levels. The observed interindividual differences match with relative ASPP2 mRNA expression levels as determined by qRT-PCR against GAPDH as a housekeeping gene (bottom of the plot for each patient).
(TIF)

Figure S2 Methylation gDNA array. Five patients with prognostic higher-risk acute myeloid or lymphoblastic leukemia were analyzed in a methylation array to determine methylation status of ASPP2. Analysis of 20 probes spanning from the transcription start site (TSS) to the 3′-untranslated region (UTR) reveal high methylation patterns - particularly in the 5′UTR and immediate downstream coding regions (15/20 probes with a threshold of ≥10%; 10/20≥50%) in all tested patients.
(TIF)
