# Supplementary figures and images for: Attenuated Expression of Apoptosis Stimulating Protein of p53-2 (ASPP2) in Human Acute Leukemia Is Associated with Therapy Failure
Source: PLoS One. 2013 Nov 27;8(11):e80193. doi: 10.1371/journal.pone.0080193 (PMC3842400; doi:10.1371/journal.pone.0080193)

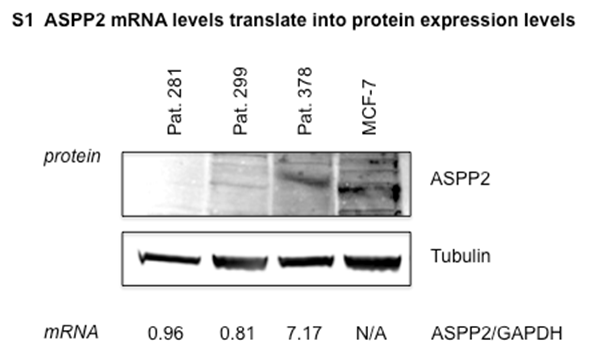

Supplement: Figure S1 — ASPP2 mRNA levels translate into protein expression levels. Protein lysates of native leukemia samples with a good-prognostic profile (patient characteristics are provided with Table 1) are immunoblotted to detect ASPP2 protein levels. The MCF-7 breast cancer cell line is used as a positive control to detect ASPP2 protein levels. The observed interindividual differences match with relative ASPP2 mRNA expression levels as determined by qRT-PCR against GAPDH as a housekeeping gene (bottom of the plot for each patient). (TIF) [file pone.0080193.s001.tif]

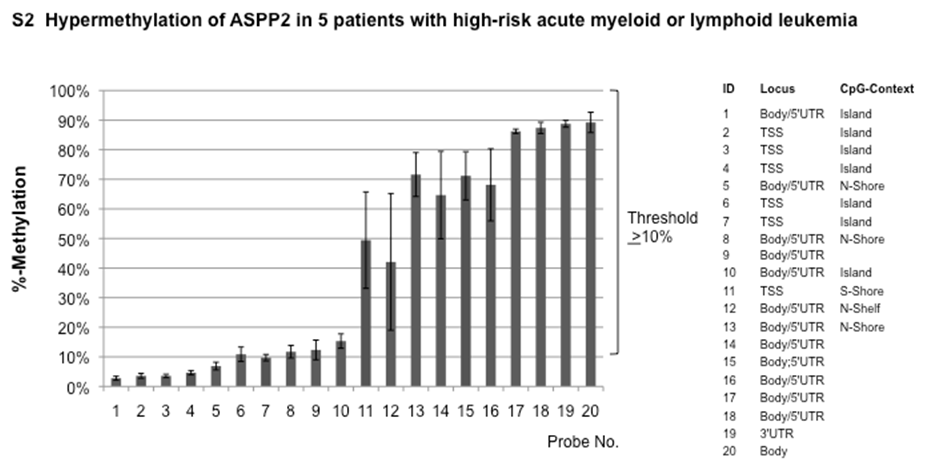

Supplement: Figure S2 — Methylation gDNA array. Five patients with prognostic higher-risk acute myeloid or lymphoblastic leukemia were analyzed in a methylation array to determine methylation status of ASPP2. Analysis of 20 probes spanning from the transcription start site (TSS) to the 3′-untranslated region (UTR) reveal high methylation patterns - particularly in the 5′UTR and immediate downstream coding regions (15/20 probes with a threshold of ≥10%; 10/20≥50%) in all tested patients. (TIF) [file pone.0080193.s002.tif]
